# Optical Absorption and Visible Photoluminescence from Thin Films of Silicon Phthalocyanine Derivatives

**DOI:** 10.3390/ma7096585

**Published:** 2014-09-12

**Authors:** Arturo Rodríguez Gómez, Carlos Moises Sánchez-Hernández, Ilán Fleitman-Levin, Jesús Arenas-Alatorre, Juan Carlos Alonso-Huitrón, María Elena Sánchez Vergara

**Affiliations:** 1 Instituto de Física, Universidad Nacional Autónoma de México, Coyoacán, 04510 México, D.F., Mexico; E-Mails: arodriguez@fisica.unam.mx (A.R.G.); jarenas@fisica.unam.mx (J.A.-A.); 2 Facultad de Ingeniería, Universidad Anáhuac México Norte, Avenida Universidad Anáhuac 46, Col. Lomas Anáhuac, Huixquilucan, Estado de México 52786, Mexico; E-Mails: cmoises.sanchez@hotmail.com (C.M.S.-H.); ifleitman@gmail.com (I.F.-L.); 3 Instituto de Investigaciones en Materiales, Universidad Nacional Autónoma de México, A. P. 70-360, Coyoacán, 04510 México, D. F., Mexico; E-Mail: alonso@unam.mx

**Keywords:** thin films, optical properties, absorption spectra, photoluminescence (PL)

## Abstract

The interest of microelectronics industry in new organic compounds for the manufacture of luminescent devices has increased substantially in the last decade. In this paper, we carried out a study of the usage feasibility of three organic bidentate ligands (2,6-dihydroxyanthraquinone, anthraflavic acid and potassium derivative salt of anthraflavic acid) for the synthesis of an organic semiconductor based in silicon phthalocyanines (SiPcs). We report the visible photoluminescence (PL) at room temperature obtained from thermal-evaporated thin films of these new materials. The surface morphology of these films was analyzed by atomic force microscopy (AFM) and scanning electron microscopy (SEM). AFM indicated that the thermal evaporation technique is an excellent resource in order to obtain low thin film roughness when depositing these kinds of compounds. Fourier transform infrared spectroscopy (FTIR) spectroscopy was employed to investigate possible changes in the intra-molecular bonds and to identify any evidence of crystallinity in the powder compounds and in the thin films after their deposition. FTIR showed that there was not any important change in the samples after the thermal deposition. The absorption coefficient (α) in the absorption region reveals non-direct transitions. Furthermore, the PL of all the investigated samples were observed with the naked eye in a bright background and also measured by a spectrofluorometer. The normalized PL spectra showed a Stokes shift ≈ 0.6 eV in two of our three samples, and no PL emission in the last one. Those results indicate that the Vis PL comes from a recombination of charge carriers between conduction band and valence band preceded by a non-radiative relaxation in the conduction band tails.

## 1. Introduction

In our days, the use of organic semiconductors for the manufacture of complex electronic devices is evident. Thousands of products based on organic light-emitting diode (OLED) technology are sold monthly. These include: televisions, mobile phones, lamps, and even displays for endoscopic imaging. Multiple studies show the diversity of molecules used as organic semiconductors and their feasibility to implement them in electronic devices [1,2,3,4,5,6,7,8,9].

In the field of organic semiconductors, the phthalocyanines (Pcs) have been pioneer molecules. They show electronic properties that make them suitable in diverse applications, from sensors [10] to organic light-emitting devices [11]. Moreover, Pcs and metal Pcs (MPcs) have been extensively studied in their optical, electrical, and structural properties [8,9,12,13,14,15,16,17,18,19,20,21,22,23,24,25,26,27,28,29,30,31,32].

From all the previous researches is worth to highlight the following: (1) Pcs, MPcs and their multiple complexes are able to absorb light within the range of 470–570 nm of the visible spectrum [20,21]; (2) many Pcs have been identified five transition bands, labeled as Q, B, N, L, and C bands, and their corresponding energies are approximately 2, 3.6, 4.4, 5.0, and 5.9 eV [20]; (3) the MPcs can be easily sublimated in order to obtain thin films without decomposition [18,23,33]; (4) depending on the deposition conditions, different molecular orientations and even film crystallinity can be obtained [34]; (5) powder MPcs exists in several crystalline polymorphs, including the α-, β-, and γ-structures [35]; and (6) in Pcs-based compounds, the efficiency and the emission energy of photoluminescence (PL) depends of polymorphic modifications [18,36,37]. And finally, MPcs derivatives thin films are able to offer a variety of optoelectronic properties by simple substitution of the central metal ion and by the modification of its periphery with suitable functional/non-functional groups, leading to many new complexes [38,39].

As seen before, Pcs have enormous modification potential. That combinatory character, gives to Pcs the relevance to be used as a basis molecule in the design of new organic semiconductor materials. It is virtually possible to tune properties like PL, light absorption energy and band gap within the natural limits of Pcs. In this paper, we synthesized three new materials based on silicon Pc (SiPc) and three organic bidentate ligands with high electronic delocalization. Those SiPc derivatives were deposited by vacuum thermal evaporation technique to obtain non-crystalline thin films.

Although Pcs field has been widely developed, there are not many studies regarding SiPcs thin films and their PL properties. Additionally, SiPc is a good research subject because there are interesting technological applications using them [40,41]. Thereupon, our main interest in this work is to report the characteristics of the optical absorptions and PL of our three new complexes based on SiPcs in order to make them available for electronic devices if further studies demonstrate their usage feasibility.

We carried out the ultraviolet-visible (UV-vis) characterization because Pcs may miss one or two of their transition bands. Therefore, the determination of their optical properties is important in designing organic optoelectronic devices [20]. Additionally, in a crystalline semiconductor the absorption spectrum terminates abruptly at the energy gap [38,39,42], however in amorphous semiconductors a tail in the absorption spectrum encroaches into the gap region. This tail makes the absorption edge of an amorphous semiconductor difficult to define experimentally. Some models have been developed to provide an approximation of the optical band gap. The Tauc, Forouhi-Bloomer, and Cody models, are most applicable for the mentioned purpose [42,43,44]. In this paper, we calculate the optical band gap using the Cody model.

For electroluminescent (EL) applications, PL properties give important reference because it is possible to estimate the EL spectra and emission efficiencies by using PL spectra and the quantum yields [27]. We performed PL measurements to confirm the emission observed with the naked eye. One of the main observed results is that for our compounds, the ligand changes the intensity but not the position of the maximum emission PL peak. The surface morphology of our deposited materials was analyzed by atomic force microscopy (AFM) and scanning electron microscopy (SEM). AFM indicated that the thermal evaporation technique is an excellent resource in order to obtain low thin film roughness an important parameter, if to fabricate a device is sought.

Finally, Fourier transform infrared spectroscopy (FTIR) measurements were conducted in both powder and thin film to identify any indication of decomposition while evaporation. FTIR showed that there was not any important change in the samples after the thermal deposition. X-ray diffraction (XRD) was carried out over the materials deposited in thin films to search for crystallinity. Any evidence of long-range crystallinity in any of these new materials was not found.

## 2. Results and Discussion

**Figure 1:**
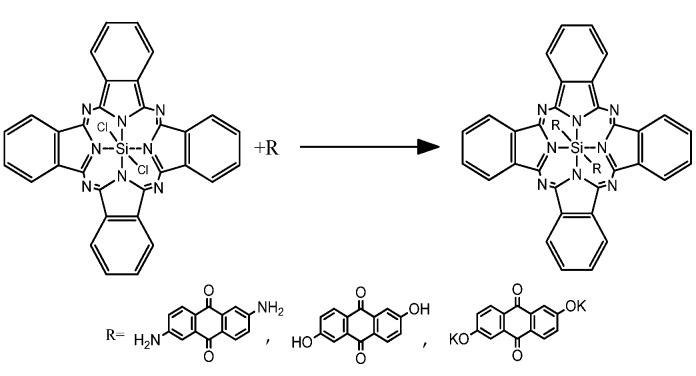
General reaction to obtain SiPcL_2_ derivatives. SiPc: silicon phthalocyanines.

This section deals with three main topics: (I) the structure; (II) the optical properties; and (III) the PL obtained from the deposited films. In order to get information on the structure of the thin films the samples were examined using infrared spectroscopy (IR), SEM and AFM. The lack of solubility in chloro ligands of SiPc molecule (SiPcCl_2_) has prevented to some extent the study of their multiple applications. Hence, novel SiPcL_2_ derivatives (Figure 1) was prepared in order to increase their solubility, which would result in a more simple characterization and a better identification of optical and semiconductor properties [40,41].

### 2.1. Structure of SiPcL_2_ Derivatives

IR spectroscopy was carried out to identify the ligand attached to the SiPcCl_2_ unit and the crystalline nature of the compounds. This is because IR spectrum of Pcs is strongly dependent on the chemical composition and the crystalline form [45,46]. Table 1 shows the signals corresponding to Pc ring [47]: two bands appearing at about 3078 ± 7 cm^−1^ and 3057 ± 1 cm^−1^ are assigned to CH asymmetric and symmetric stretching vibrations in the ring and those bands at 2936 ± 1 cm^−1^ and 2803 ± 2 cm^−1^ are assigned as the CH symmetric and asymmetric stretching vibrations as alkyl. The band appearing at 1612 ± 1 cm^−1^ was assigned to the C–C stretching in isoindole. The band appearing at 683 ± 2 cm^−1^ was assigned the C–C macrocycle ring deformation. While the band appearing at 1118 ± 3 cm^−1^ is assigned to the C–H bending in plane deformations [48]. Additionally, the two bands appearing at 910 ± 4 cm^−1^ and 728 ± 6 cm^−1^ were assigned to C-H bending out of plane deformations. It is noteworthy to observe the bands appearing at 1288 ± 1, 1164 ± 1 and 1077 ± 2 cm^−1^, that were assigned to the C–N in isoindole in plane band in pyrrole stretching vibration, respectively [48]. From Table 1, it is possible to notice that the materials exhibit C=O and C–O functional groups with wavelengths 1660 cm^−1^ and 1070 cm^−1^, respectively. Other important bands are the NH_2_ (related to an amine for Compound **A**) located in the 3210 cm^−1^ to 3421 cm^−1^ interval. The presence of the N–H vibrational band may indicate that bidentate amines are coordinated at their ends. It could also be possible that two ligands are coordinated to one single molecule of the silicon macrocycle. According to Kumar* et al.* [49] and Busch* et al.* [50], on nucleophilic additions of bidentate amines to metallic macrocycles, an infrared absorption band located in the 3945–3374 cm^−^^1^, region is associated with a N–H vibration.

**Table 1:** Characteristic Fourier transform infrared spectroscopy (FTIR) bands for KBr pellets and thin films (cm^−1^). MPc: metal phthalocyanines; and SiPcCl_2_: chloro ligands of SiPc molecule.

MPc	ν (CH) cm^−1^	ν (C–C) cm^−1^	ν (C–N) cm^−1^	ν (C=O) cm^−1^	ν (C–O) cm^−1^	α-form cm^−1^	β-form cm^−1^
Compound **A**: pellet	3068, 3056, 2934, 2806, 1116, 900, 732	1617, 685	1291, 1163	1663	1075	-	781, 734
Compound **A**: thin film	3073, 3056, 2937, 2805, 1121, 908, 734	1613, 681	1288, 1164	1663	1078	-	-
Compound **B**: pellet	3079, 3062, 2934, 2800, 1122, 912, 732	1606, 685	1291, 1163	1664	1075	720	781, 737
Compound **B**: thin film	3071, 3058, 2937, 2801, 1118, 907, 723	1612, 684	1288, 1165	1662	1076	-	-
Compound **C**: pellet	3079, 3056, 2934, 2800, 1116, 906, 731	1612, 679	1285, 1163	1664	1076	728	782, 735
Compound **C**: thin film	3085, 3056, 2935, 2801, 1120, 1077, 908, 735	1611, 683	1289, 1165	1662	1077	-	-
SiPcCl_2_: pellet	3087, 3059, 1116, 2937, 2801, 907, 718	1612, 684	1286, 1160	-	1073	-	781, 737
SiPcCl_2_: thin film	3085, 3058, 1115, 2937, 2803, 908, 720	1612, 682	1289, 1165	-	1076	-	-

**Figure 2:**
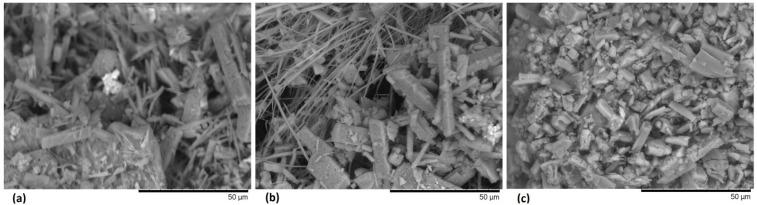
Scanning electron microscopy (SEM) micrographs of: (**a**) Compound **A**; (**b**) Compound **B**; and (**c**) Compound **C** films at 1500×.

The main spectral features distinguishing the different crystalline forms of MPc were found to lie in the region of 800–700 cm^−1^. These spectral differences are attributed to the different crystalline stacking of the MPcs molecules, especially those around 731 cm^−1^ and 777 cm^−1^ for the β-form and those around 720 cm^−1^ for the α-form [51]. From these studies, we are able to determine the phase and any significant chemical changes, which may occur in these materials during the thermal evaporation. In Table 1, can be observed that originally, SiPcCl_2_ was present in crystalline β structure. By adding the different bidentates ligands, Compound **A** maintained the same crystalline structure, however, Compounds **B** and **C**, initiated a transformation to the α phase (724 ± 4 cm^−1^) that was not completed. The above can be proved from the remaining signals at 736 ± 2 cm^−1^ and 781 ± 1 cm^−1^, referent to β phase. The difference between these two phases is the angle formed between the symmetry axis and the stacking direction. Alpha crystals have angles of 26.5°, while beta crystals involve angles of 45.8°. Although the bidentate ligand has similar structure, the functional groups at the extremes are the responsible of the crystalline change. Apparently, the acid character of the anthraflavic and its derivative salt, generate molecules orientations in different directions to the amine groups. Figure 2 shows the SEM images for three compounds. In the images it is possible to observe structures with regular geometry, as well as the growth of crystalline needles with sharp angles (Figure 2a,b), obtained during the purification of the compounds.

On the other hand, when these materials were deposited over the silicon substrate at 298 K, a transition to an amorphous-form took place. We conclude the above because IR spectroscopy studies do not show the signs correspondent to α and β phases. It is important to note that the evaporation of tetravalent Pc powder forms an amorphous thin film [52]. Apparently, the thermal gradient between layer temperature and the compound temperature is high enough to avoid the nucleation and the growth of ordered structures in thin films. FTIR showed that there was not any important change in the samples after the thermal deposition. Table 1 shows the main signals for these compounds in both KBr pellets and thin film. The deposited films are formed by the same macro-ions than those of the original synthesized compounds. Thus, the locations of the absorption bands in the spectra of the synthesized powders and the deposited films are nearly the same. When deposited on thin films, the signals from our compounds show slight changes. This is due there are internal stresses that lead to little variations in angles and bonding energies. However, there is no significant change due to evaporation. After annealing at 473 K and oven cooling-down, the IR spectrum does not show important changes for thin films.

**Figure 3:**
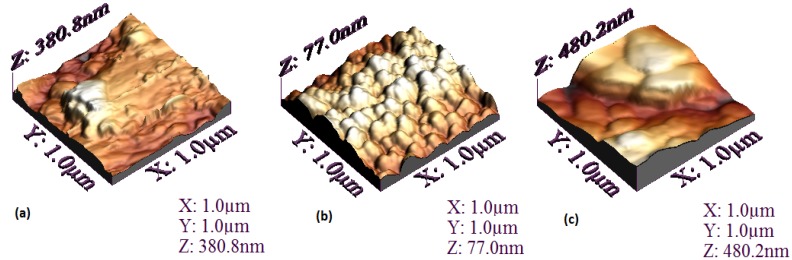
Atomic force microscopy (AFM) images for: (**a**) Compound **A**; (**b**) Compound **B**; and (**c**) Compound **C** films deposited on Q-substrate.

Figure 3 shows AFM images of the SiPcL_2_ films at room temperature. From the AFM images is possible to note that the chemical structure of the complex is what defines the aggregation state on the substrate. The size and distribution of the grains, as well as the roughness, change for each SiPcL_2_ derivative, although all of them show compact deposits. Thin film from Compound **B** (Figure 3b) is homogeneous and contains a fine granular structure. In the case of films from Compounds **A** and **C**, a heterogeneous distribution is seen and particles agglomerate to generate irregular structures (Figures 3a–c). Further studies must be done to determine if those morphological variations observed in our thin films can be related to other important physical properties.

The calculated root mean square (RMS) roughness for the thin films are shown in Table 2. The difference in the roughness values may be related to the different bidentate ligand in each SiPcL_2_ derivative. The final molecule obtained with the substitution of each ligand is different in size and molecular weight, and some properties such sublimation point and nucleation easiness also change. As would be expected according to the AFM image shown in Figure 3b, the minor wrinkle is for Film **B**, synthesized from anthraflavic acid. Apparently the size of the hydroxile group compared to the size of the substitutes in the anthraquinone molecules of Compounds **A** and **C** (amino and potassium group, respectively), promotes the homogeneous stacking and less rugosity. On the other hand, although the deposit conditions are practically similar for the three films, the thickness measured (see Table 2), is minor for Compound **A**. The above, is a consequence of the greater chemical stability in the molecule generated by the primary amines of Compound **A**, a vacuum was required in a higher magnitude order for the deposit of this film, yet, its thickness is considerably less than those obtained for Films **B** and **C**.

**Table 2:** Characteristic parameters of thin-films under investigation. RMS: root mean square.

Sample	RMS roughness (nm)	Refractive index (*n*)	% Reflectance	Thickness (nm)	Cody *Eg* ^a^ (eV)
Compound **A**	69.19	2.022	11.44	542	1.7, 2.1, 3.1
Compound **B**	14.53	2.621	20.04	1319	1.6, 2.5
Compound **C**	110.42	2.696	21.11	1562	1.6, 2.4

^a^ *Eg*: band gap.

### 2.2. Optical Properties

The refractive indices (*n*) of the films, obtained from ellipsometric measurements are shown in Table 2, as well as the reflectance percentages at normal incidence. The refraction index is practically the same for Films **B** and **C**, and different for Compound **A**. Similar results are obtained by comparing the reflectance, which for these type of thin films apparently depend on the structure of the ligand. The amino group in Compound **A**, has a slightly distorted tetrahedral geometry with the nitrogen at the center. The optical properties of the film could be modified by the binding of the metal atom with the molecule and by the nonbonding electron pair. There is an inversion of the nitrogen atom due to the delocalization of its free electrons in the aromatic ring. Therefore, there is a decrease in the refractive index and reflectance compared to Compounds **B** and **C**, where oxygen atoms are responsible for the binding.

**Figure 4:**
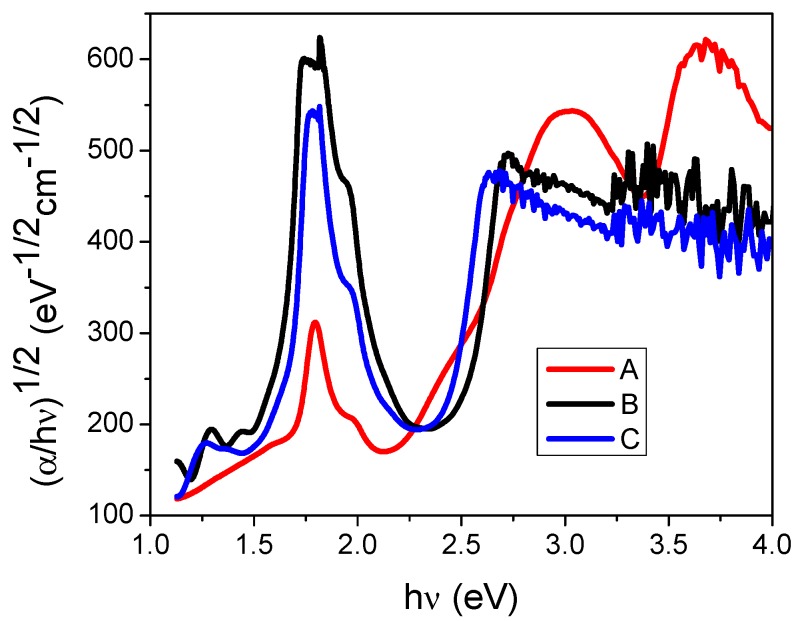
Plot of (α/*h*ν)^1/2^* vs.* photon energy *h*ν of thin Films **A**, **B** and **C** deposited on Q-substrate.

The Cody model is used for the determination of the optical gap. Even though this model has been mainly used for inorganic materials, it can also be employed for organic compounds with reliable results. The model is based on the measurement of optical absorption at the ultraviolet (UV)/visible range [42,43,44]. Cody model provides an effective and easy option for the determination of the optical band of thin films, it uses the dependence between the photon energy and the absorption coefficient. Additionally, the use of this model is suggested in dichotic systems, such as SiPcL_2_. The dichotic systems are conformed by the union of the planar molecule and the ligand and they are able to organize in monodimensional columns that can transport charges efficiently in just one direction. In this model, it is assumed that the disorder responsible for relaxing the momentum conservation rules should also influence the underlying distribution of electronic states [42,43,44]. In accordance with this model, the optical gap associated with SiPcL_2_ thin films should rather be determined by extrapolating the linear trend observed in the spectral dependence of (α*/h*ν)^1/2^ = f(*h*ν) [42,43,44,53], over a limited range of photon energies. The abscissa axis intersection of this linear extrapolation corresponds to the Cody optical gap (Figure 4) of the film, the Cody optical gap values are contained in Table 2. It is important to remark that according to Figure 4 and Table 2, the analysis of the spectral behaviour for Compounds **A**–**C** reveals similar indirect allowed transitions around 1.6 eV in the beginning of the absorption spectrum and 2.3 ± 2 eV for the fundamental energy gap. The band gap does not show any remarkable difference from its value, this may be attributed to the morphology that have the same system and differ only in the structure and size of the bidentate ligand and the arrangement of the molecules. Additionally, the gap depends on the number of electron in the outer shell form metal cation [51,54] and for Films **A**–**C** is the same. The energy gaps of 1.6 eV and 2.3 eV, could correspond to a transition involving the valence band (highest occupied molecular orbital, HOMO) to the bottom of the conduction band (lowest unoccupied molecular orbital, LUMO) and to the next unoccupied orbital. For Compound **A**, the high-energy peak at 3.1 eV may correspond to transition involving the valence band to the split d band [51]. Possibly, for this compound, there is a bigger electronic movement and less steric hindrance between the MPc and the ligand. Silicon with an oxidation state +4 has a hard Lewis acid behavior. Although it is reduced by the macrocycle, silicon binds with the ligand amino group. In turn, it exhibits a hard Lewis base behavior, and its electronic density is polarized in mayor manner than in the cases of the ligands in Compounds **B** and **C**. There, the hydroxy group and its potassium salt, generate electrostatic bonds, opposite to the acid-base interaction in Compound **A** that shows a significant covalent component.

**Figure 5:**
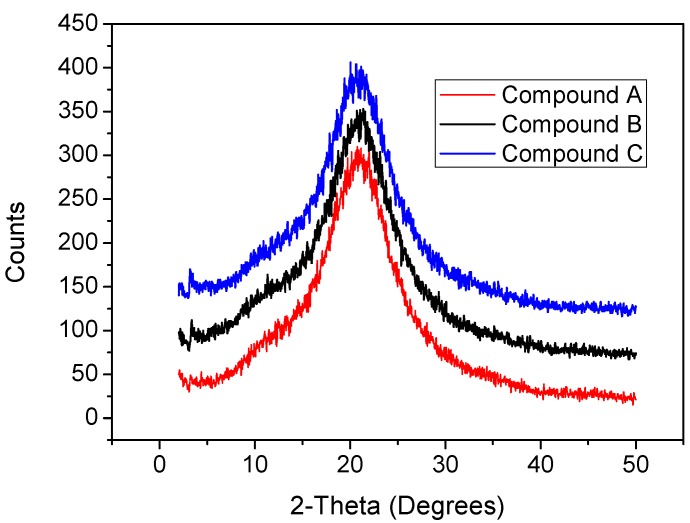
X-ray diffraction (XRD) pattern of thin Films **A**, **B** and **C**.

On the other hand, the absorption spectrum of the α-form has a doublet around 1.75 eV and 1.9 eV, while in the tricyclic spectrum of the β-form two shoulders are observed at 2 eV and 2.2 eV [55]. In the optical spectrum (Figure 4) for Film **A**, no signals of the crystalline forms are shown, indicating the presence of an amorphous structure. The optical spectrum for deposited thin Films **B** and **C** showed the small doublet at 1.8 eV and 1.95 eV, as corresponding to the β-form, possibly combined with an amorphous structure, according with IR results. For the purpose of detecting whatever signs of crystalline structures, the characterization by means of XRD was undertaken on the deposited materials. Figure 5 shows an XRD trace of the as-deposited SiPc derivatives thin film.

As it may be observed, there is only one shallow peak at approximately 2θ = 3.415° and 3.229° for thin Films **B** and **C** respectively implying amorphous structure and the presence of some degree of crystallinity [51]. All this indicates that, while several phtalocyanine films have been reported as crystalline polymorphs [48,51,52], the vacuum evaporation technique gave rise to desalination in the atomic structure. During the deposition process, when the molecules reach that portion of the substrate at the lowest temperature, their kinetic energy does not suffice in order for them to possess a high-enough surface mobility. Therefore, the long-reach order, characteristic of crystals, is not achieved and an amorphous film structure results.

### 2.3. Photoluminescence Measurements

**Figure 6:**
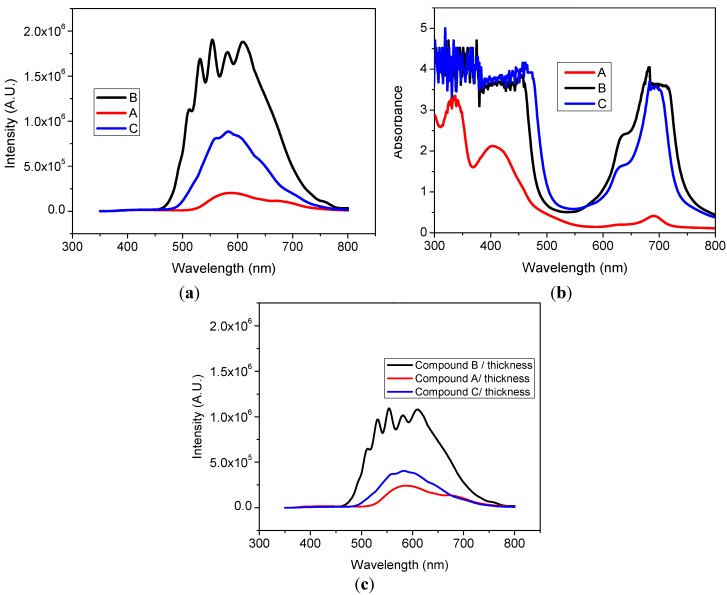
(**a**) Photoluminescence (PL); and (**b**) absorbance spectra of thin Films **A**, **B**, and **C**; (**c**) PL normalized to the thickness of each film.

Figure 6a,b shows the PL and absorbance spectra of the tree films, obtained at room temperature. As can be seen, the intensity of the PL of Sample **B** is higher than that of Sample **C**, and the lowest intensity correspond to Sample **A**. This behavior was consistent with the fact that intense PL in the visible range was observed with the naked eye only for Films **B** and **C**, but it was not perceived for Sample **A**. Since the different sublimation temperatures of each material lead to different film thicknesses, even using the same evaporation parameters, and, in addition, conscious that the PL intensity depends on the sample thickness. We carried out the normalization of the PL spectra to the thickness of each film. This normalization is also useful to make a fair comparison of the influence of each ligand to the PL efficiency. Figure 6c shows this normalization. It is noteworthy that Compound **A** exhibits some PL but due its lower thickness it cannot be perceived with the naked eye. It is also important to note that the total PL intensity increases when the antraflavic acid is the ligand (Compound **B**) and decreases for the amine ligand (Compound **A**).

The variation of the PL signal as a function of position on the sample was minimal, which was consistent with the uniformity found for the thickness and refractive index of the films on the entire substrate [56]. The PL emitted by the excitation spot on the films appeared as follows: light yellow (Compound **B**), dark yellow (Compound **C**), and with an intensity high enough to be observed with the naked eye against a bright background.

The PL spectra of the Sample **B** shows two main central peaks and two side shoulders that may be a result from an interference effect due to the bigger film thickness [57,58]. The absorbance (*A*) spectra of the films (Figure 6b) show optical absorption peaks and edges distributed in the wavelength range from infrared to UV. The absorption spectrum for MPcs originates from molecular orbital within the aromatic 18π electron system and from overlapping orbitals on the central metal atom [59]. The UV-Vis spectra of SiPcL_2_ exhibited characteristic Q and B bands, one in the visible region at 600–700 nm (Q-band) attributed to the π → π* transition from the HOMO to the LUMO of the Pc^2−^ ring, and the other in the UV region at 300–400 nm (B-band) arising from the deeper π levels → LUMO transitions [60]. The Q-band is split into two distinct peaks in the visible region due to molecule aggregation or molecular distortion [51].

As observed from Figure 6b, for Compounds **B** and **C**, the Q-band consists of a high-energy peak around 680 nm and a low energy shoulder at 630 nm, thin Film **A** contains only the high-energy peak at 690 nm [40,41,54]. The high-energy peak of the Q-band has been assigned to the first π–π* transition on the Pc macrocycle. The low-energy peak of the Q-band has been explained either, as a second π–π* transition, as an excitation peak, as a vibrational internal interval and as a surface state [51]. The Q-band is associated to the ligand coordination to the silicon ion in the ring. Electrons are, therefore, able to transfer energy throughout the structure and become responsible for the absorption spectra [51]. An examination of the Soret band or the B-band in the UV region reveals one peak around 335 nm for all the thin films [40,41,54]. B-band of the SiPcL_2_ films, arising from the deeper π levels → LUMO transitions [60]. The observation of a single peak in Soret band is similar to that observed for CoPc, NiPc, and other Pcs [35,54,61]. This may imply that the splitting structure of this peak could be affected by the orbital overlapping of Pc ring with the central metal [61]. The values of the absorbance bands depend on the film thickness according to Beer’s lay [54]. The smaller thickness is for thin Film **A**, which also has the lowest absorbance (see Figure 6b).

Differently from other studies made for crystalline trivalent or tetravalent MPcs [62], our SiPc films do not show long range order. This is due to the deposition conditions, which are fundamental in thin film fabrication. Thermal gradient, substrate roughness, evaporation velocity, and even initial nucleation points, are factors that must be carefully studied to be able to make crystalline depositions. Therefore is important to state that due the XRD diffractogram showed not any long-range order in our thin films and, therefore, the PL properties are not related to crystallinity of the materials after deposition. Figure 5 shows the diffractograms of our three compounds. Compounds **B** and **C** are possible to distinguish a short range order with a very small peak at low angles.

**Figure 7:**
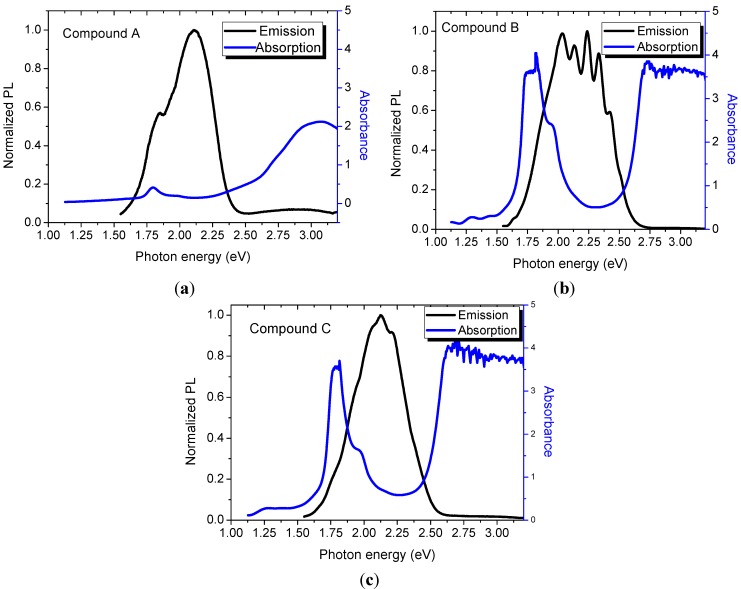
Normalized PL and absorbance* vs.* photon energy of thin films: (**a**) Film **A**; (**b**) Film **B**; and (**c**) Film **C**.

In order to give a first quick explanation on mechanisms of the visible PL and the relationship with the optical absorption and electronic structure of these thin films, the PL, and absorbance spectra of Figure 7 were plotted together in an overlapping scheme in terms of photon energy. Since the analyzed samples had different thicknesses (see Table 2) and the PL integrated intensity depends upon this parameter, the PL spectra of Figure 5c was normalized to the thickness of each sample. In Figure 7, the PL was normalized to maximum intensity because our main priority in this figure is to observe the absorbing-emitting regions. At first glance, we can identify three important features. First, the samples that exhibit naked eye PL (**B** and **C**) have an absorption valley located at almost the same wavelength as the maximum emission peak. For Sample **B** the absorption is at 2.75 eV and emission at 2.12 eV. While Sample **C** is absorbing at 2.75 eV and emitting at 2.23 eV. Second, the width of the emission and absorption spectra is the same for Samples **B** and **C** ≈ 0.7 eV. Finally, note that Sample **A** shows no intense PL but it has a weak absorption peak at 2.8 eV. In our three samples the photon absorption is originated between the Bands B and Q. The amorphous nature of the thin films allows the apparition of multiple delocalized states between HOMO and LUMO. Delocalized states promote a non-radiative relaxation and, therefore, a Stokes shift around 0.6 eV.

The energy of maximum PL peak is related to the optical band gap. In Pcs, as well as other planar cyclic molecules, π electron delocalization is the main responsible for the semiconducting properties [63]. The molecular organization in our compounds is probable to be similar to the sheet arrangement proposed by Yang [32]. The bidentate ligands used to synthesize the compounds promote a better conformation of the sheet arrangement, but SiPcs are the most responsible of the electronic properties of the final new materials. Therefore, a big change is observed in PL efficiency and a despicable change in maximum PL peak when different ligand is used.

According to our data, the PL in Pc thin films is substantially affected by the intramolecular structure. Samples **B** and **C** have a Stokes shift, 0.63 eV and 0.52 eV, respectively. Sample **A** exhibits no emission and nor absorption at 2.75 eV. The above is a marker of the excitation-emission mechanism present in these materials. Ligands used in Samples **B** and **C** (anthraflavic acid and potassium derivative salt of anthraflavic acid) are able to generate a compound with a well-defined optical gap. In these compounds, the Stokes shift is due to the localized states in the tails of the bands. Those localized states generate non-radiative transitions (relaxations) of the carriers. Once the carriers reach the edge of the conduction band, they recombine to the valence band emitting a photon with the remaining energy after relaxation. Is noteworthy that the band tails of Compound **C** are smaller than in Compound **B** and hence the Stokes shift is also smaller. Meanwhile, thin Film **A** is plagued with localized defects in its optical gap that prevent the PL emission.

It is most important to note that the absorptions in Bands B and Q, which can be observed in our compounds, are completely related to the SiPcs and not to the ligand. However, the observed differences in the absorption values could be affected by the arrangement of the molecules [62]. In this vein, we could presume that ligands must be affecting to the electronic and optical properties of the final molecules, not by adding or removing localized states, but modifying the final arrangement of the molecule.

## 3. Experimental Section

### 3.1. Synthesis

#### 3.1.1. Starting Material and Chemicals

The raw materials for this work were obtained from commercial sources with no purification prior to their use. In this case, 2,6-diaminoanthraquinone (C_14_H_10_N_2_O_2_), 2,6-dihydroxyanthraquinone (C_14_H_8_O_4_) and its potassium derivative salt were used in order to form a substitution between the organic compound and the labile axial SiPcCl_2_ [40].

#### 3.1.2. Synthesis of Compound **A**

0.3 g (1.26 mmol) of C_14_H_10_N_2_O_2_ was dissolved in 60 mL of methanol. Then add 0.30 g (0.49 mmol) of phthalocyaninato-dichlorosilane (C_32_H_16_Cl_2_N_8_Si). The resulting blue powder was recrystallized and dried at high vacuum, yielding 55% of the reaction. Melting point (m.p.): 300 °C. MS(FAB^+^, DMSO/EtOH) *m*/*z*: 236 [C_14_H_8_N_2_O_2_]^+^, 540 [C_32_H_16_SiN_8_]^+^, 575 [C_32_H_16_ClSiN_8_]^+^, 776 [C_46_H_24_SiN_10_O_2_]^+^.

#### 3.1.2. Synthesis of Compound **B**

0.3 g (1.25 mmol) of anthraflavic acid (C_14_H_8_O_4_) and 0.30 g (0.49 mmol) of C_32_H_16_Cl_2_N_8_Si were dissolved. The resulting green powder was recrystallized and dried at high vacuum, yield 49%. M.p.: 300 °C. MS(FAB^+^, DMSO/EtOH) *m*/*z*: 239 [C_14_H_7_O_4_]^+^, 540 [C_32_H_16_SiN_8_]^+^, 575 [C_32_H_16_ClSiN_8_]^+^, 779 [C_46_H_23_SiN_8_O_4_]^+^.

#### 3.1.3. Synthesis of Compound **C**

Follow a similar procedure as for Compounds **A** and **B**, using 0.3 g (0.95 mmol) of potassium derivative salt of anthraflavic acid (C_14_H_6_O_4_K_2_) and 0.30 g (0.49 mmol) of C_32_H_16_Cl_2_N_8_Si. The resulting blue powder was recrystallized and dried at high vacuum, yield 53%. M.p.: 300 °C. MS(FAB^+^, DMSO/EtOH) *m*/*z*: 277 [C_14_H_6_O_4_K]^+^, 540 [C_32_H_16_SiN_8_]^+^, 575 [C_32_H_16_ClSiN_8_]^+^, 817 [C_46_H_22_SiN_8_O_4_K]^+^.

### 3.2. Powder Characterization

The IR spectroscopy characterization of powdered materials was carried out by means of a Nicolet iS5-FT spectrophotometer (Waltham, MA, USA) using KBr pellets for solid samples within the 4000 cm^−1^ to 300 cm^−1^ region with an 8 cm^−1^ resolution. Mass spectra (FAB+) were measured on a 3-nitrobenzyl alcohol support in the positive ion mode on a Jeol JMS-SX102A instrument (Tokyo, Japan).

### 3.3. Thin-Film Deposition

A vacuum chamber operated by an arrangement of mechanical-diffusion pumps and a special fabricated molybdenum crucible with a double-grid cover were used to carry out the depositions. The operation pressure used in the evaporation process was 1 × 10^−4^ Torr for compounds with C_14_H_8_O_4_ and its potassium derivative salt, and 1 × 10^−5^ Torr for compound with amine. The crucible-substrate distance was 20 cm. 1 g of quartz fiber was placed above the Pc powders inside the crucible to avoid the ejection of grains towards the substrate at a temperature of 563 K. All depositions were made on high resistivity (200 Ω·cm) single crystalline silicon wafer n-type (100) (Si-substrate) and quartz substrates (Q-substrate). Q-substrate was ultrasonically degreased in 1,2-dichloroethane and methanol, later dried with a high-pressure flux of nitrogen gas. Meanwhile, Si-substrate was chemical etched for 5 min with a *p* solution (10 mL HF, 15 mL HNO_3_ and 300 mL H_2_O) in order to remove the native oxide from the c-Si surface. All the samples were annealed at a temperature 473 K for 2 h in air.

### 3.4. Thin-Film Characterization

SEM was carried out in a Hitachi microscope. FT-IR spectroscopy measurements were obtained with a spectrophotometer Nicolet iS5-FT using Si-substrate. UV-Vis spectra, *Unicam* spectrophotometer, Model UV300 (Waltham, MA, USA), in the range of 190–1100 nm, with a 0.5 nm resolution (films on Q-substrate). The XRD analysis was performed with the θ–2θ technique using a Bragg-Brentano Rigaku ULTIMA-IV Diffractometer (Tokyo, Japan) and working with CuKα (λ = 0.15405 nm) radiation over a quartz substrate. Ellipsometry, *Gaertner Scientific Corporation* Ellipsometer, Model L117 (Skokie, IL, USA), with a He-Ne laser operating at 632.8 nm and an incidence angle of 70° (films deposited on Si-substrate). Profilometry, *Dektak* profilometer, Model IIA (films evaporated on Q-substrates, Plainview, NY, USA). PL measurements were carried out in a dark room at room temperature, using a beam (λ = 325 nm, 25 mW) from a Kimmon He-Cd Laser (Tokyo, Japan) as excitation source, with an incident angle of 45° relative to the normal of the sample. The luminescence was collected at an angle normal to the sample using a quartz optic fiber and measured in the range from 350 nm to 800 nm with a Fluoromax-Spex Spectrofluorometer (Edison, NJ, USA). For PL, infrared and ellipsometric measurements the substrates used were (100) oriented and 200 Ω·cm CSi slices.

## 4. Conclusions

The organic compounds used in microelectronics had had a boom in the last ten years. The main advantages of the organic luminescent devices are the mechanical flexibility and energy efficiency. However, it is necessary to continue synthesizing and depositing thin films on new luminescent organic materials because the existing ones are not enough to meet the demands of the microelectronics industry. In this paper, the experimental procedure for the synthesis of a soluble silicon(IV) Pc derivatives is reported. Originally, SiPcCl_2_ exhibits a crystalline β structure. When adding the different bidentates ligands, Compound **A** maintained the same crystalline structure, however, Compounds **B** and **C** initiated a transformation to the α phase that was not completed. The evaporation of tetravalent Pc powders leads to the formation of amorphous thin films, and, after annealing, does not show important changes for thin Film **A**, and the crystallization to the β-form is induced in thin Films **B** and **C**, possibly combined with an amorphous structure. In Compounds **B** and **C**, intense PL was observed with the naked eye. The absorption and photoemission spectra let us deduce that visible PL comes from a recombination of charge carriers between conduction band and valence band preceded by a non-radiative relaxation in the conduction band tails. In this paper, a methodology of synthesis and deposition of two new materials that might be used in the design of EL devices is presented. Our new materials could be incorporated to the microelectronics industry if in the future a more specialized study is able to show good EL efficiency.
